# Association of Cytokine IL-17, IL-4, IL-6, and IL-12 Gene Polymorphisms in Rheumatoid Arthritis Patients in a Tertiary Care Hospital in Bangladesh

**DOI:** 10.1155/2024/3728179

**Published:** 2024-02-02

**Authors:** Taskin Jahan, Ahmed Abu Saleh, Shaheda Anwar

**Affiliations:** Department of Microbiology and Immunology, Bangabandhu Sheikh Mujib Medical University, Dhaka, Bangladesh

## Abstract

Rheumatoid arthritis (RA) is a chronic autoimmune inflammatory disease that involves cytokines in its pathogenesis. This study is aimed at investigating if gene polymorphisms in cytokines like IL-17, IL-4, IL-6, and IL-12 affect RA susceptibility and severity in the Bangladeshi population. This was a cross-sectional comparative study that included 40 diagnosed RA patients according to the American College of Rheumatology (ACR) criteria 2010, who were free from other rheumatological diseases, and 40 healthy subjects for comparison. The study used PCR-RFLP to determine the IL-17, IL-4, IL-6, and IL-12 cytokine gene polymorphisms. Patients had a mean age of 37.22 ± 6.70 years. Among the patients, 31 were female and 9 were male. The mean disease duration was 18.11 ± 7.39 months. The study found that rheumatoid arthritis patients with the IL-17F (7488 A/G) polymorphism with GG genotype (*P* = 0.006, OR = 8.56, 95% CI = 1.77 − 41.33) and IL-12B (1188 A/C) polymorphism with AC (*P* = 0.012, OR = 3.69, 95% CI = 1.43 − 9.53) and CC (*P* = 0.013, OR = 7.58, 95% CI = 1.56 − 36.88) genotypes were significantly associated with disease risk. Furthermore, patients with the IL-17F (7488) GG genotype and IL-12B (1188) AC and CC genotypes had higher rheumatoid arthritis disease severity and activity parameters. The study found no significant association between polymorphisms involving IL-4 (590 C/T) and IL-6 (174 G/C) genes and rheumatoid arthritis disease risk in the Bangladeshi population. Gene polymorphisms in cytokines IL-17F (7488 A/G) and IL-12B (1188 A/C) can predict disease susceptibility and severity in Bangladeshi rheumatoid arthritis patients.

## 1. Background

Rheumatoid arthritis (RA) is a common systemic inflammatory autoimmune disease that causes synovial inflammation and can result in chronic and irreparable joint degeneration. Multiple factors, including genetic, environmental, and hormonal factors, contribute to the development of certain autoimmune processes [1]. According to the global prevalence of RA, Australia, North America, and Western Europe (0.4% to 0.5%) have a higher prevalence of RA than Asia, the Middle East, and North Africa (0.16%) [2]. In Bangladesh, the prevalence of rheumatoid arthritis is 1.6%, with women having a much greater frequency (2.4%) than men (0.7%) [3]. Genetic predisposition is associated with a 50–60% risk of RA [4]. Among non-HLA genetic factors, gene polymorphisms, particularly cytokines and their receptors, have an essential role in RA pathogenesis [5].

IL-17 is a proinflammatory cytokine that is made up of six ligands and five receptors [6]. Among the six ligands, IL-17A and IL-17F have key functional and biological features, and the genes encoding these two cytokines are found on human chromosome 6p12.2 [7, 8]. Chabaud et al. first suggested the role of IL-17 in RA development by demonstrating the presence of this cytokine in the synovial fluid of RA patients [9]. IL-17 participates in tissue inflammation and degradation by promoting the production of metallo matrix proteases and other proinflammatory cytokines, including tumor necrosis factor-*α* (TNF-*α*), IL-1, and IL-6, which attract neutrophils, macrophages, and lymphocytes to the synovium [10]. There is a histidine-to-arginine substitution at amino acid 161 in the 3rd exon of the IL-17F gene in the IL-17F 7488 A/G polymorphism [11]. The IL-17F (7488 A/G) polymorphism has been shown to be associated with RA in some previous studies [12–14].

The cytokine IL-4 modulates a wide range of immunological processes, including immunoglobulin isotype switching, class- II MHC expression by B cells, and the differentiation of certain T-cell subsets [15]. C-to-T base replacement is represented by the IL-4-590 promoter polymorphism, which is located at 589 base pairs (bp) before the transcriptional site [16]. The IL-4 gene polymorphism (-590 T/C) has been linked to an increased risk of RA in European and Chinese populations [17, 18].

Many RA patients have high levels of IL-6 in their blood and synovial fluid. IL-6 induces inflammation and joint degeneration by acting on neutrophils, which produce reactive oxygen intermediates and proteolytic enzymes [19]. Polymorphisms involving the IL-6 (-174 G/C) gene with a G-to-C substitution at position -174 have been linked to an increased risk of RA in the Chinese Han population [18].

IL-12 is a proinflammatory cytokine that increases the production of IFN-*γ*, which is responsible for the differentiation of naive T cells into Th1 cells. It also increases the cytotoxicity of natural killer cells and cytotoxic T lymphocytes [20]. Many RA patients' blood and synovial fluid contain higher levels of IL-12 [21]. The IL-12B (+1188 A/C) polymorphism was found to increase susceptibility to RA in the Chinese population [22, 23].

Genetic polymorphisms usually differ between ethnic groups. Polymorphisms in the IL-17, IL-4, IL-6, and IL-12 genes may contribute to RA pathogenesis and act as a risk or protective factor for the disease. Polymorphisms involving IL-17, IL-4, IL-6, and IL-12 have been shown to be a risk for RA in some Asian countries, including China and Pakistan. In our country, polymorphisms of these cytokines have not yet been studied in RA patients.

### 1.1. Study Objective

The aim of this study was to determine if there was a link between IL-17, IL-4, IL-6, and IL-12 gene polymorphisms and rheumatoid arthritis disease susceptibility and severity in the Bangladeshi population, which might help in understanding whether these cytokine polymorphisms act as risk factors for the Bangladeshi rheumatoid arthritis population and may help in future treatment development.

### 1.2. Research Question

Is there any association of cytokine IL-17, IL-4, IL-6, and IL-12 gene polymorphisms with disease susceptibility and severity in rheumatoid arthritis patients in the Bangladeshi population?

## 2. Methods

### 2.1. Study Design and Patient Selection

This study was a cross-sectional comparative analysis involving 40 patients diagnosed with rheumatoid arthritis according to the 2010 American College of Rheumatology (ACR) criteria [24] and 40 healthy subjects for comparison. All patients were recruited from the Department of Rheumatology, Bangabandhu Sheikh Mujib Medical University (BSMMU), from January 2023 to March 2023.

The exclusion criteria for patients were as follows: (a) patients under 18 years of age and age more than 60 years, (b) patients with other autoimmune diseases, and (c) patients suffering from major illnesses, such as hepatic failure, renal failure, and cancer.

Age and sex-matched 40 healthy subjects were selected from resident doctors, teachers, and laboratory staffs of the Department of Microbiology and Immunology, BSMMU, without any diagnosed autoimmune or rheumatological disease.

### 2.2. Sample Size

Sample size for this study was calculated by using the following formula (two-tailed *Z*-test):

(1) $$\begin{array}{c} n=\frac{{\left\{u\sqrt{\pi o\left(1-\pi o\right)+\pi 1\;\left(1-\pi 1\right)}+v\sqrt{2\bar{\pi }\left(1-\bar{\pi }\right)}\right\}}^{2}}{{\left(\pi 1-\pi o\right)}^{2}}, \end{array}$$

where *n* is the estimated sample size, *πo* is 66% which is the proportion for group 1 (this is the patient or experimental group [13]), *π*1 is 92% which is the proportion for group 2 (this is reference or healthy subject's group [13]), *π*1 − *πo* is the difference to be detected by the study, *u* is 1.96 (in 95% confidence interval), and *v* is 0.84.

Therefore, the estimated sample size was

(2) $$\begin{array}{c} n=\frac{{\left\{1.96\sqrt{0.66\left(1-0.66\right)+0.92\left(1-0.92\right)}+0.84\sqrt{2\times 0.79\left(1-0.79\right)}\right\}}^{2}}{{\left(0.92-0.66\right)}^{2}}=35. \end{array}$$

The sample size was taken 40, due to available sample.

So, the sample size was 40 in each group.

### 2.3. Selection Bias

Based on the exclusion and inclusion criteria, patients and healthy subjects were selected randomly. A structured data format was prepared to collect necessary and similar data from all the patients to exclude data collection bias.

### 2.4. Variables

Dependent variables were as follows: ACR score, DAS28, and HAQ.

Independent variables were as follows: SNP of IL-17 F (+7488 A/G), IL-4 (-590 C/T), IL-6 (-174 G/C), and IL-12B (+1188A/C) genes, RF, anti-CCP, ESR, and CRP.

### 2.5. Patient Data

Relevant data were taken from the patients, including age, sex, disease duration, and number of tender and swollen joints. Rheumatoid arthritis disease severity was assessed by the Disease Activity Score 28 (DAS28). Functional status was assessed by the Health Assessment Questionnaire (HAQ). Patient laboratory investigations, including erythrocyte sedimentation rate (ESR), C-reactive protein (CRP), rheumatoid factor (RF), and anti-cyclic citrullinated peptide antibodies (anti-CCP) were recorded.

### 2.6. Detection of Gene Polymorphisms by PCR-RFLP

#### 2.6.1. DNA Extraction

DNA extraction was performed from EDTA-containing blood tubes using the Genomic DNA Extraction Spin Kit v2 (Anatolia Geneworks, Bosphore, Turkey). Extracted DNA was stored at -20°C until use.

#### 2.6.2. Genotyping Using PCR-RFLP

Polymerase chain reaction- restriction fragment length polymorphism (PCR-RFLP) was used to analyze gene polymorphisms of cytokine IL-17F (7488 A/G), IL-4 (590 C/T), IL-6 (174 G/C), and IL-12 (1188 A/C) in all subjects.

A total volume of 25 *μ*l was used for PCR comprising 15 *μ*l of master mix (Emerald Amp MAX PCR Master Mix, Takara Bio, Japan), 1 *μ*l forward primer, 1 *μ*l reverse primer, 3 *μ*l nuclease-free water, and 5 *μ*l extracted DNA.

Amplification of the polymorphism region was performed using the following primers: For IL-17F (7488 A/G), forward primer 5′-GTGTAGGAACTTGGGCTGCATCAAT-3′ and reverse primer 5′-AGCTGGGAATGCAAACAAAC-3′ were used [13]. For IL-4 (590 C/T), forward primer 5′-ACTAGGCCTCACCTGATACG-3′ and reverse primer 5′-GTTGTAATGCAGTCCTCCTG-3′ were used [18]. For IL-6 (174 G/C), forward primer 5′-GCCTCAATGACGACCTAAGC-3′ and reverse primer 5′-TCATGGGAAAAT CCCACATT-3′ were used. [25]. For IL-12B (1188A/C), forward primer 5′-CTGATCCAGGATGAAAATTTG-3′ and reverse primer 5′-CCCATGGCAACTTGAGAGCTGG-3′ were used [26].

Thermal cycling parameters for IL-17F (7488A/G) were initial denaturation at 94°C for 3 min; 35 cycles at 94°C for 30 sec, 60°C for 30 sec, and 72°C for 30 sec; and a final extension at 72°C for 7 min [12]. Initial denaturation at 94°C for 5 min, followed by 40 cycles at 94°C for 30 sec, 57°C for 30 sec, and 72°C for 35 sec, and a final extension at 72°C for 10 min were the thermal cycling parameters for IL-4 (590 C/T) [18]. Initial denaturation at 95°C for 5 min, followed by 35 cycles at 95°C for 30 sec, 61°C for 30 sec, and 72°C for 30 sec, and a final extension at 72°C for 10 min were the thermal cycling parameters for IL-6 (174 G/C) [25]. Initial denaturation at 95°C for 15 min, followed by 35 cycles at 94°C for 45 sec, 54°C for 60 sec, and 72°C for 60 sec, and a final extension at 72°C for 10 min were the thermal cycling parameters for IL-12 (1188 A/C) [26].

After amplification, PCR products were digested by 0.5 *μ*l NIaIII, 1 *μ*l BsmFI, 0.5 *μ*l NlaIII, and 1 *μ*l TaqI-V2 restriction enzymes for genes IL-17 F (+7488 A/G), IL-4 (-590 C/T), IL-6 (-174G/C), and IL-12B (+1188A/C), respectively.

**Figure 1:**
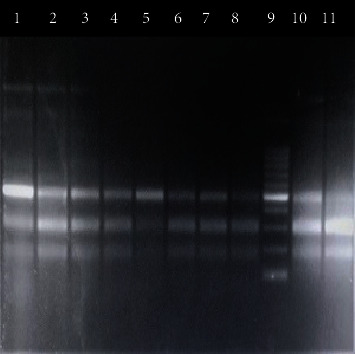
PCR-RFLP of IL-17 (+7488 A/G) SNP by NIaIII restriction enzyme. Lane 5 shows GG genotype (418 bp), lanes 8 and 11 show AA genotype (288 bp, 130 bp), and lanes 1, 2, 3, 4, 6, 7, and 10 show AG genotype (418 bp, 288 bp, 130 bp). Lane 9 shows a 100 bp DNA ladder.

**Figure 2:**
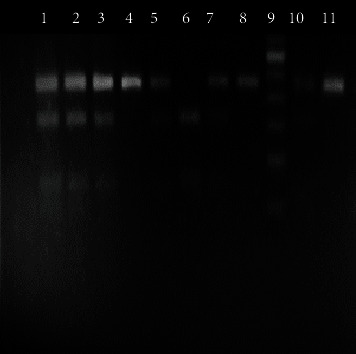
PCR-RFLP of IL-12B (+1188 A/C) SNP by TaqI-V2 restriction enzyme. Lanes 4, 5, 7, 8, 10, and 11 show AA genotypes (226 bp), lanes 1, 2, and 3 show AC genotypes (226 bp, 155 bp, 71 bp), and lane 6 shows CC genotype (155 bp, 71 bp). Lane 9 shows a 50 bp DNA ladder.

**Figure 3:**
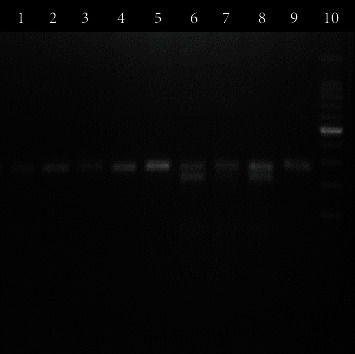
PCR-RFLP of IL-4 (590 C/T) SNP by BsmFI restriction enzyme. Lanes 1, 2, 3, 4, 5, and 9 show TT genotypes (252 bp), and lanes 6, 7, and 8 show CT genotypes (252 bp, 192 bp). Lane 10 shows a 100 bp DNA ladder.

**Figure 4:**
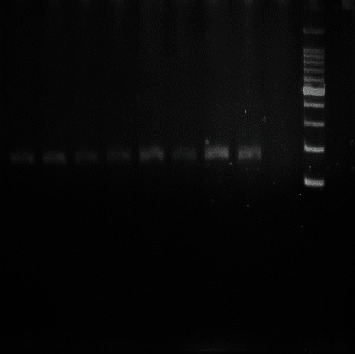
PCR-RFLP of IL-6 (-174 G/C) SNP by NIaIII restriction enzyme. All lanes show GG genotypes (163 bp).

**Figure 5:**
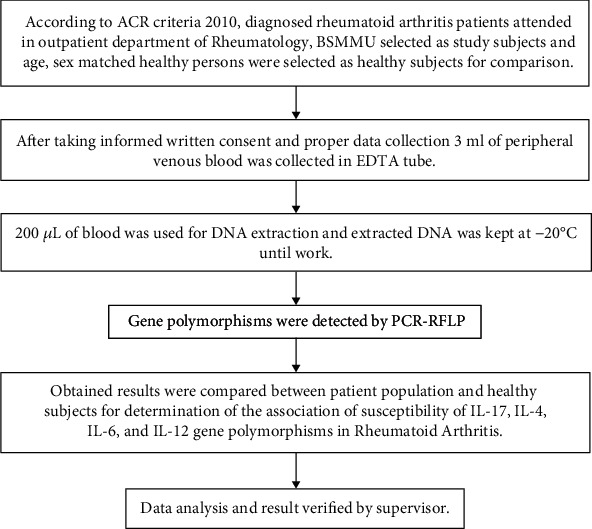
Workflow diagram.

The digested product was subjected to electrophoresis in a 2% agarose gel stained with ethidium bromide. After electrophoresis, DNA bands were visualized under UV light (Figures 1–4). A work flow diagram has been shown in Figure 5.  Table 1 shows the amplicon sizes before and after digestion.

### 2.7. Statistical Analysis

The SPSS software package version 27 (Strata Corporation, College Station, Texas) was used to analyze the data. Continuous variables were expressed as mean, standard deviation, and median. Categorical variables were expressed as frequency and percentage. The chi-square (*χ*^2^) test was used to assess the significant differences in genotype and allele frequencies between the two research groups. The paired *t*-test was used to examine the association between polymorphisms and disease activity and severity measures. When the *P* value was < 0.05, it was considered statistically significant.

### 2.8. Expected Outcomes

If cytokine IL-17F (7488 A/G), IL-4 (590 C/T), IL-6 (174 G/C), and IL-12 (1188 A/C) gene polymorphisms became statistically significant in RA patients in comparison to healthy subjects, it might be said that these polymorphisms act as risk factor for RA disease pathogenesis in Bangladeshi population.

## 3. Results

This study included 40 clinically confirmed cases of rheumatoid arthritis patients and 40 healthy subjects. Rheumatoid arthritis patients had a mean age of 37.22 ± 6.70 years. Among the patients, 31 were female (77.5%) and 9 were male (22.5%). The male-female ratio was 1 : 3.4. Mean disease duration was 18.11 ± 7.39 months. Mean value of ESR was 41.17 (5-120) mm in the 1^st^ hour, CRP -14.40 (0.75-71.9) mg/l, RF-163.90 (9.39-615) IU/ml, and anti-CCP -138.75 (0.7-422) U/ml. RF was positive in 36 patients (90%) and negative in 4 patients (10%); anti-CCP was positive in 37 patients (92.5%) and negative in 3 patients (7.5%). The mean values of DAS28 and HAQ were 4.29 ± 1.23 and 0.82 ± 0.67, respectively. According to the DAS28, 3 patients (7.5%) were in remission; 8 patients (20%) were in low disease activity; 18 patients (45%) were in moderate disease activity; and 11 patients (27.5%) were in severe disease activity. According to HAQ, mild disability was in 26 (65%) patients, moderate disability was in 10 (25%), and severe disability was in 4 (10%) patients (Table 2).

All participated patients were on methotrexate. But dose, duration of drugs, and their effect on genotype distribution were not assessed in this study.

Table 3 shows the statistically significant differences in genotype distribution and allele frequency of IL-17F (7488 A/G) and IL-12B (1188 A/C) polymorphisms between rheumatoid arthritis patients and healthy subjects. For the IL-17F (7488A/G) polymorphism, a significant association was found between the homozygous mutant GG genotype and RA susceptibility (*P* = 0.006, OR = 8.56, 95% CI = 1.77 − 41.33). G allele was significantly different between patients and healthy subjects. Patients carrying the G allele were 3.67 times more at risk of developing RA than healthy subjects (*P* = 0.039, OR = 3.67, 95% CI = 1.90 − 7.07). The homozygous AA genotype was found to be a protective factor for RA in this study. For the IL-12B (1188A/C) polymorphism, the homozygous mutant CC genotype and heterozygous mutant AC genotype were significantly associated with an increased risk of RA. Patients carrying the CC genotype had a 7 times higher risk of RA development than healthy subjects (*P* = 0.013, OR = 7.58, 95% CI = 1.56 − 36.88). Patients carrying the AC genotype were 3 times more at risk of RA development than healthy subjects (*P* = 0.012, OR = 3.69, 95% CI = 1.43 − 9.53). The mutant C allele was significantly different between patients and healthy subjects and was associated with increased RA risk (*P* = 0.023, OR = 2.11, 95% CI = 1.10 − 4.04). Patients with the homozygous AA genotype were protected from RA risk.

There were no significant differences in the genotype and allele frequency of the IL-4 (590C/T) and IL-6 (174G/C) gene polymorphisms between RA patients and healthy subjects. None had the homozygous CC genotype for the IL-4 (590C/T) polymorphism, and no subjects had the GC and CC genotype for the IL-6 (174G/C) polymorphism.

Disease activity and severity measures of rheumatoid arthritis were substantially (*P* < 0.05) higher in the IL-17F (7488 A/G) GG genotype than in the AA genotype (Table 4) and in the IL-12B (1188 A/C) AC and CC genotypes than in the AA genotype (Table 5).

## 4. Discussion

Rheumatoid arthritis is a multifactorial disease, and multiple genes may interact and influence disease susceptibility, severity, chronicity, and immune response [29]. As genetic factors, genetic polymorphisms in cytokines and their receptors can play roles in RA pathogenesis. The associations of polymorphisms in the cytokine genes IL-17F (7488A/G), IL-4 (590C/T), IL-6 (174G/C), and IL-12B (1188A/C) with RA disease susceptibility and severity were assessed in this study.

In this study, the proportion of females and males were 77.5% and 22.5%, respectively. The male-female ratio was 1 : 3.4. Nearly similar findings were present in other studies that included China (female, 76.7%; male, 23.3%; M : F, 1 : 3.3) and Tunisia (female, 80.5%; male, 19.5%; M : F, 1 : 4.1) [13, 22].

This study found that IL-17F (7488A/G) polymorphism with homozygous mutant GG genotype was found to be a significant risk for RA patients (*P* = 0.006, OR = 8.56, 95% CI = 1.77 − 41.33). Patients with the mutant GG genotype had 8.56 times higher risk of RA than healthy subjects. The mutant G allele frequency was significantly (*P* = 0.039) higher in RA patients than in healthy subjects. Similar findings were found in a study in Tunisia that showed that GG and AG genotypes were associated with RA risk (*P* < 0.0001), and the mutant G allele distribution was significantly higher in patients than in controls (*P* = 0.00002) [13]. A Polish study also found discernible differences in the genotype distribution and allele frequency of the IL-17F (7488A/G) polymorphism between RA patients and healthy controls [12]. In this study, the RA disease parameters DAS28 (5.16 ± 0.89), HAQ (1.12 ± 0.67), RF (268.3 ± 156.5 IU/ml), and anti-CCP (241.2 ± 115.6 U/ml) were significantly (*P* < 0.05) increased in patients with the GG genotype compared to those with the AA genotype. A study conducted in Poland was similar to this finding, where RA patients with the IL-17F GG genotype had more severe disease (DAS28 > 5.1) [12]. A study in Brazil found no association between the IL-17F (7488A/G) polymorphism and RA disease parameters [27].

In this study, there were substantially more homozygous mutant CC genotypes and heterozygous AC genotypes in RA patients than in healthy subjects for the IL-12B (1188A/C) polymorphism. Patients with the CC genotype had a 7.58 times higher risk of RA development, and patients with the AC genotype had a 3.69 times higher risk of RA. The prevalence of the mutant C allele was significantly higher in RA patients than in healthy subjects. These findings were similar to a study in China, where the genotype frequencies of AC+CC of IL-12B (1188 A/C) significantly varied between RA patients and healthy controls, and the frequency of the C allele was significantly higher in RA patients (*P* ≤ 0.001) [23]. Here, patients with AC and CC genotypes had a significant association with RA severity compared to the AA genotype (*P* < 0.05). A Chinese study found that the IL-12B (1188A/C) AC/CC genotype was a significant risk factor for RA in RF-positive patients (*P* = 0.04), but they did not find any significant association of the IL-12B AC/CC genotype with DAS28 and HAQ score [22].

Polymorphisms involving the IL-4 (590 C/T) and IL-6 (174 G/C) genes were not associated with RA risk in this study. A similar finding was found in a study in Poland for the IL-4 (590 C/T) polymorphism [30] and a study in Iraq for the IL-6 (174 G/C) polymorphism [31]. They did not find these polymorphisms to be risk factors for RA. A study in China showed that IL-4 (590 C/T) and IL-6 (174 G/C) gene polymorphisms were risk factors for RA [18].

## 5. Conclusion

The study concluded that the IL-17F (7488 A/G) polymorphism with GG genotype and G alleles is related to the severity and susceptibility to rheumatoid arthritis. The IL-12B (1188A/C) polymorphism with genotypes CC and AC and C alleles is related to the susceptibility and severity of RA disease. The probability of developing rheumatoid arthritis illness was not correlated with the IL-4 (590 C/T) or IL-6 (174 G/C) gene polymorphisms in this study.

## 6. Limitations

This was a single center-based study with a small sample size, so the result may not represent whole RA patients in Bangladesh. Effects of treatment on genotype distribution were not considered in this study; it would be better if considered.

## Figures and Tables

**Table 1:** Amplicon size after digestion.

Genes	Amplicon size before digestion	Amplicon size after digestion	References
IL-17F (7488 A/G)	412 bp	AA = 288, 124 bp AG = 412, 288, 124 bp GG = 412 bp	[27]

IL-4 (590 C/T)	252 bp	CC = 192, 60 bp CT = 192, 60, 252 bp TT = 252 bp	[28]

IL-6 (174G/C)	163 bp	GG = 163 bp GC = 163, 111, 52 bp CC = 111, 52 bp	[25]

IL-12B (1188A/C)	226 bp	AA = 226 bp AC = 226, 155, 71 bp CC = 155, 71 bp	[26]

**Table 2:** Rheumatoid arthritis patients' clinical and laboratory characteristics (*n* = 40).

Characteristics	Cases (*n* = 40)
Age (years), mean ± SD (range)	37.22 ± 6.70 (20-50)
Gender	
Male	9 (22.5%)
Female	31 (77.5%)
Male : female	1 : 3.4
Disease duration (months)	18.11 ± 7.39
Number of tender joints, mean (range)	12.17 (0-28)
Number of swollen joints	3.10 (0-14)
ESR (mm in the 1^st^ hour)	41.17 (5-120)
CRP (mg/l)	14.40 (0.75-71.9)
RF (IU/ml)	163.90 (9.39-615)
Anti-CCP (U/ml)	138.75 (0.7-422)
DAS28, mean ± SD	4.29 ± 1.23
DAS28, *n* (%)	
Remission (≤2.6)	3 (7.5%)
Low (2.6-≤3.2)	8 (20%)
Moderate (3.2-≤5.1)	18 (45%)
Severe (>5.1)	11 (27.5%)
HAQ, mean ± SD	0.82 ± 0.67
HAQ, *n* (%)	
Mild disability	26 (65%)
Moderate disability	10 (25%)
Severe disability	4 (10%)
RF positive patients, *n* (%)	36 (90%)
RF negative patients, *n* (%)	4 (10%)
Anti-CCP-positive patients, *n* (%)	37 (92.5%)
Anti-CCP-negative patients, *n* (%)	3 (7.5%)

**Table 3:** Comparison of genotype distribution and allele frequencies of IL-17F (7488 A/G), IL-12B (1188 A/C), IL-4 (590C/T), and IL-6 (174 G/C) polymorphisms in patients with rheumatoid arthritis and healthy subjects (*n* = 80).

Genotype and allele	Patients (*n* = 40)	Healthy subjects (*n* = 40)	OR (95% CI)	*P* value
IL-17F (7488A/G)				
AA	3 (7.5%)	18 (45%)	0.09 (0.02-0.34)	0.019^∗^
AG	25 (62.5%)	20 (50%)	1.66 (0.68-4.04)	0.650
GG	12 (30%)	2 (5%)	8.56 (1.77-41.33)	0.006^∗^
G allele	49 (61.2%)	24 (30%)	3.67 (1.90-7.07)	0.039^∗^
A allele	31 (38.8%)	56 (70%)		
IL-12B(1188A/C)				
AA	7 (17.5%)	28 (70%)	0.09 (0.03-0.26)	0.001^∗^
AC	22 (55%)	10 (25%)	3.69 (1.43-9.53)	0.012^∗^
CC	11 (27.5%)	2 (5%)	7.58 (1.56-36.88)	0.013^∗^
C allele	38 (47.5%)	24 (30%)	2.11 (1.10-4.04)	0.023^∗^
A allele	42 (52.5%)	56 (70%)		
IL-4 (590C/T)				
CC	—	—	—	—
CT	25 (62.5%)	15 (37.5%)	2.78 (1.12-6.87)	0.05
TT	15 (37.5%)	25 (62.5%)	0.36 (0.15-0.89)	0.05
T allele	55 (68.7%)	65 (81.3%)	0.50 (0.24-1.04)	0.195
C allele	25 (31.3%)	15 (18.7%)		
IL-6 (174 G/C)				
GG	40	40		
GC	—	—		
CC	—	—		

*P* value was determined by chi-square test; ^∗^*P* value < 0.05 indicates significance.

**Table 4:** Effect of IL-17F (7488 A/G) polymorphism on RA disease severity and activity.

Disease parameters	Genotypes		
	AA (*n* = 3)	AG (*n* = 25)	GG (*n* = 12)
Disease severity parameters			
HAQ	0.13 ± 0.00	0.76 ± 0.65^∗^	1.12 ± 0.67^∗^
DAS28	3.23 ± 0.74	4.99 ± 0.82^∗^	5.16 ± 0.89^∗^
RF	15.9 ± 0.26	157.2 ± 244.94^∗^	268.3 ± 156.5^∗^
Anti-CCP	65.53 ± 72.9	122.15 ± 76.5^∗^	241.2 ± 115.6^∗^
Disease activity parameters			
ESR	10.0 ± 0.0	42.48 ± 26.58^∗^	50.71 ± 23.37^∗^
CRP	2.89 ± 2.75	14.2 ± 14.9^∗^	24.02 ± 22.85^∗^
Number of tender joints	8.66 ± 9.86	12.16 ± 9.21^∗^	13.71 ± 8.19^∗^
Number of swollen joints	0.33 ± 0.57	2.9 ± 3.6^∗^	5.14 ± 4.29^∗^

^∗^ *P* value < 0.05 indicates significance.

**Table 5:** Effect of IL-12B (1188 A/C) polymorphism on RA disease severity and activity.

Disease parameters	Genotypes		
	AA (*n* = 7)	AC (*n* = 22)	CC (*n* = 11)
Disease severity parameters			
HAQ	0.4 ± 0.37	1.11 ± 0.70^∗^	0.51 ± 0.47^∗^
DAS28	3.27 ± 1.17	4.66 ± 1.07^∗^	4.56 ± 1.15^∗^
RF	100.3 ± 136.5	184 ± 196.6^∗^	188.6 ± 194.83^∗^
Anti-CCP	23.99 ± 45.99	169.35 ± 103.26^∗^	117.86 ± 79.93^∗^
Disease activity parameters			
ESR	22.43 ± 16.71	46.2 ± 26.0^∗^	42.91 ± 27.96^∗^
CRP	3.67 ± 2.42	19.13 ± 18.71^∗^	11.79 ± 12.37^∗^
Number of tender joints	7.6 ± 8.3	13.36 ± 8.38^∗^	14.45 ± 9.46^∗^
Number of swollen joints	0.14 ± 0.38	3.5 ± 3.2^∗^	4.18 ± 5.01^∗^

*P* value was determined by paired *t*-test; ^∗^*P* value < 0.05 indicates significance.

## Data Availability

The corresponding author can provide data that are analyzed in this study on request.

## Abbreviations

RA: Rheumatoid arthritis
OR: Odds ratio
CI: Confidence interval
SD: Standard deviation
ESR: Erythrocyte sedimentation rate
CRP: C-reactive protein
DAS-28: Disease Activity Score in 28 Joints
HAQ: Health Assessment Questionnaire
RF: Rheumatoid factor
Anti-CCP: Antibodies to cyclic citrullinated peptide
EDTA: Ethylenediaminetetraacetic acid
SNP: Single nucleotide polymorphism.
